# Evaluation of Antifungal Activity and Mode of Action of New Coumarin Derivative, 7-Hydroxy-6-nitro-2H-1-benzopyran-2-one, against *Aspergillus* spp.

**DOI:** 10.1155/2015/925096

**Published:** 2015-06-14

**Authors:** Felipe Queiroga Sarmento Guerra, Rodrigo Santos Aquino de Araújo, Janiere Pereira de Sousa, Fillipe de Oliveira Pereira, Francisco J. B. Mendonça-Junior, José M. Barbosa-Filho, Edeltrudes de Oliveira Lima

**Affiliations:** ^1^Pharmaceutical Science Department, Federal University of Paraíba, 58051-970 João Pessoa, PB, Brazil; ^2^Laboratory of Synthesis and Drug Delivery, Biological Science Department, State University of Paraíba, 58020-540 João Pessoa, PB, Brazil; ^3^Center of Education and Heath, Federal University of Campina Grande, 58175-000 Cuité, PB, Brazil

## Abstract

*Aspergillus* spp. produce a wide variety of diseases. For the treatment of such infections, the azoles and Amphotericin B are used in various formulations. The treatment of fungal diseases is often ineffective, because of increases in azole resistance and their several associated adverse effects. To overcome these problems, natural products and their derivatives are interesting alternatives. The aim of this study was to examine the effects of coumarin derivative, 7-hydroxy-6-nitro-2H-1-benzopyran-2-one (Cou-NO_2_), both alone and with antifungal drugs. Its mode of action against *Aspergillus* spp. Cou-NO_2_ was tested to evaluate its effects on mycelia growth and germination of fungal conidia of *Aspergillus* spp. We also investigated possible Cou-NO_2_ action on cell walls (0.8 M sorbitol) and on Cou-NO_2_ to ergosterol binding in the cell membrane. The study shows that Cou-NO_2_ is capable of inhibiting both the mycelia growth and germination of conidia for the species tested, and that its action affects the structure of the fungal cell wall. At subinhibitory concentration, Cou-NO_2_ enhanced the *in vitro* effects of azoles. Moreover, in combination with azoles (voriconazole and itraconazole) Cou-NO_2_ displays an additive effect. Thus, our study supports the use of coumarin derivative 7-hydroxy-6-nitro-2H-1-benzopyran-2-one as an antifungal agent against *Aspergillus* species.

## 1. Introduction

The incidence and prevalence of invasive fungal infections have increased in recent years, especially in the currently large population of immunocompromised patients and those hospitalized with serious underlying diseases. Fungal species represent 25% of the microorganisms isolated in blood cultures of hospitalized patients. Of these, species of the genus* Aspergillus* spp. have the highest incidence among the filamentous fungi [1, 2].

*Aspergillus* spp. produce a wide variety of diseases. The main route of infection is penetration by air. In cases of invasive aspergillosis* Aspergillus fumigatus* is the most common species isolated in the world. In Brazil, the species* A. flavus* is the most common [3]. The main clinical manifestations observed due to* Aspergillus* spp. infections are cutaneous aspergillosis, otomycosis, aspergilloma, and sinusitis [4].

For the treatment of such infections, the azoles (Fluconazole, Itraconazole, and Voriconazole) and Amphotericin B are used in various formulations. However, with the increase of azole resistance, and the several adverse effects associated with the use of Amphotericin B (which include nephrotoxicity and neurotoxicity [5]), the treatment of fungal diseases is often ineffective, which has caused alarm among health professionals.

To overcome these problems, natural products and their derivatives are interesting alternatives. The coumarins (phenolic compounds which possess a benzopyranone nucleus and are one of the major classes of secondary metabolites) have been highlighted in antimicrobial activity studies [6–8].

**Figure 1:**
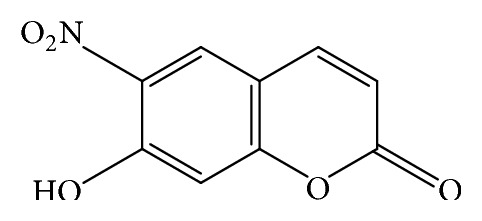
Chemical structure for 7-hydroxy-6-nitro-2H-1-benzopyran-2-one (Cou-NO_2_).

Recently reported by our group, the antifungal activity against* Aspergillus fumigatus* and* A. flavus* of twenty-four coumarin derivatives was described. Some of these derivatives showed significant antifungal activity with Minimum Inhibition Concentration (MIC) values ranging from 16 to 32 *μ*g/mL. 7-hydroxy-6-nitro-2H-1-benzopyran-2-one (Figure 1) revealed an MIC value of 16 *μ*g/mL [9]. Despite the promising results of the study, a need remains for better assessment of the effects of coumarin derivatives on the fungus structure and possible mechanisms action.

Increases in the availability of antifungal compounds have induced searches for better therapeutic strategies, such as the use of two or more antifungal drugs in combination [10]. Combination therapy of antifungal drugs with natural products and their derivatives has been little explored; this is especially true for the coumarin derivatives, which promise an alternative against strains of* Aspergillus* spp.

The aim of this study was to examine the effects and mode of action of coumarin derivative, 7-hydroxy-6-nitro-2H-1-benzopyran-2-one, both alone and together with antifungal drugs, against* Aspergillus* spp.

## 2. Material and Methods

### 2.1. Microorganisms

*Aspergillus* spp. used in the antifungal assay were obtained from the archival collection of the Federal University of Paraíba Laboratory of Mycology (LM). They included* A. fumigatus* (ATCC 46913, LM 121, LM 743, and LM 135) and* A. flavus* (ATCC 16013, LM 35, LM 36, and LM 23). Stock inoculators (suspensions) of* Aspergillus* spp. were prepared from 8-day old potato dextrose agar (Difco Lab., USA), the cultures grown at room temperature. Fungal colonies were covered with 5 mL of sterile saline solution (0,9%), the surface was gently agitated with vortexes, and fungal elements with saline solution were transferred to sterile tubes. Inoculator was standardized at 0.5 tube of McFarland scale (10^6^ CFU/mL). The final concentration confirmation was done by counting the microorganisms in a Neubauer chamber [11–13].

### 2.2. Chemicals

The product tested was the coumarin derivative, 7-hydroxy-6-nitro-2H-1-benzopyran-2-one (Cou-NO_2_), obtained by biosynthesis [9]. Amphotericin B, Fluconazole, Itraconazole, and Voriconazole were obtained from Sigma Aldrich, Brazil. The drugs were dissolved in DMSO (dimethylsulfoxide), and sterile distilled water was used to obtain solutions of 1024 *µ*g/mL for each antifungals. The concentration of DMSO did not exceed 0.5% in the assays.

### 2.3. Culture Media

To test the biological activity of the products, Potato Agar (AP) and Sabouraud dextrose agar (SDA) were purchased from Difco Laboratories (Detroit, MI, USA), and RPMI-1640-L-glutamine (without sodium bicarbonate) (Sigma-Aldrich, São Paulo, SP, Brazil) culture media were used. They were prepared and used according to the manufacturers' instructions.

### 2.4. Minimum Inhibitory Concentration (MIC)

The determination of minimum inhibitory concentration against ATCC strains was demonstrated in an article previously published by our group [9]. We also carried out determinations of the coumarin derivative CIM in clinical strains of* A. flavus* and* A. fumigatus*. Broth microdilution assays were used to determine the MICs of coumarin derivative 7-hydroxy-6-nitro-2H-1-benzopyran-2-one (Cou-NO_2_), and Amphotericin B. RPMI-1640 was added to all the wells of 96-well plates. Twofold serial dilutions of the three agents were prepared to obtain concentrations varying between 4 *μ*g/mL and 1024 *μ*g/mL. Finally, 10 *μ*L aliquots of the inoculate suspension were added to the wells, and the plates were incubated at 28°C for 3 days. Negative controls (without drugs) were used to confirm conidia viability, and sensitivity controls (for DMSO) were also included in the studies. At 72 h there were visual observations for fungal growth. The MIC was defined as the lowest concentration capable of visually inhibiting fungal growth by 100%. The results were expressed as the arithmetic mean of three experiments [14, 15].

### 2.5. Effects on Mycelia Growth

Analysis of the interference of coumarin derivative, 7-hydroxy-6-nitro-2H-1-benzopyran-2-one, on mycelia growth was performed by determining the dry mycelia weight of* A. fumigatus* (ATCC 46913) and* A. flavus* (ATCC 16013) [15, 16]. Flasks containing MIC (16 *μ*g/mL) and MIC × 2 (32 *μ*g/mL) of coumarin derivative in RPMI-1640 medium were inoculated with suspension of the test* A. fumigatus* (ATCC 46913) and* A. flavus* (ATCC 16013) strains. In the corresponding control, the same amount of coumarin derivative was replaced by distilled water. The system was incubated at 28°C for 8 days. Flasks containing mycelia were filtered through Whatman Grade 1 Qualitative Filtration Paper (particle retention: 11 *μ*m) and then washed with distilled water. The mycelia were dried at 60°C for 6 h and kept at 40°C overnight. The filter paper containing dry mycelia from two independent assays was weighed, and the mean values were obtained. Percentage growth inhibition based on the dry weight of each at time of analysis was calculated according to Sharma and Tripathi [15].

### 2.6. Conidial Germination Assay

The coumarin derivative, 7-hydroxy-6-nitro-2H-1-benzopyran-2-one (Cou-NO_2_), and Amphotericin B were tested to evaluate effects on the germination of fungal conidia of* A. fumigatus* (ATCC 46913) and* A. flavus* (ATCC 16013). Flasks containing MIC (16 *μ*g/mL) and MIC × 2 (32 *μ*g/mL) of coumarin derivative and a control with distilled water were used. In sterile test tubes, 500 *µ*L of RPMI-1640 plus the Cou-NO_2_ were evenly mixed with 500 *µ*L of fungal conidia suspension and immediately incubated at 28°C. Samples of this mixture were taken after 24 h of incubation for analysis. The whole experiment was performed in duplicate, where the number of conidia was determined in a Neubauer chamber, and the inhibition percentage of spore germination at each time point was calculated by comparing the results obtained in the test experiments with the results of the control experiment. The analysis was conducted under an optical microscope (Zeiss Primo Star) [17, 18].

### 2.7. Sorbitol Assay Effects

The assay was performed using medium with and without sorbitol (control), to evaluate possible mechanisms involved in the antifungal activity of the test product on the* Aspergillus* spp. cell wall. The sorbitol was added to the culture medium in a final concentration of 0.8 M. The assay was performed by microdilution method in 96-well plates in a “U” (Alamar, Diadema, SP, Brazil) [11, 12]. The plates were sealed aseptically, incubated at 28°C, and readings were taken at 3 days. Based on the ability of sorbitol to act as a fungal cell wall osmotic protective agent, the higher MIC values observed in the medium with added sorbitol compared to the standard medium suggest the cell wall as one of the possible cell targets for the product tested [19–21]. Amphotericin B was used as the control drug. The assay was performed in duplicate and expressed as the geometric mean of the results.

### 2.8. Ergosterol Binding Assay: MIC Value Determination in Presence of Ergosterol

To assess if the product binds to the fungal membrane sterols, an experiment was performed according to the method described by Escalante et al. [22], with some modifications. The ergosterol was prepared as was described by Leite et al. [21]. The MIC of coumarin derivative, 7-hydroxy-6-nitro-2H-1-benzopyran-2-one (Cou-NO_2_), against* Aspergillus* spp. was determined by using broth microdilution techniques [11, 12], in the presence and absence of exogenous ergosterol (Sigma-Aldrich, São Paulo, SP, Brazil) added to the assay medium, in different lines of the same microplate. Briefly, a solution of Cou-NO_2_ was doubly diluted serially with RPMI-1640 (volume = 100 *μ*L) containing plus ergosterol at a concentration of 400 *μ*g/mL. A volume of 10 *μ*L yeast suspension (0,5 McFarland) was added to each well. The same procedure was realized for Amphotericin B, whose interaction with membrane ergosterol is already known, serving as a control drug. The plates were sealed and incubated at 28°C. The plates were read after 3 days of incubation, and the MIC was determined as the lowest concentration of test agent inhibiting the visible growth. The assay was carried out in duplicate and the geometric mean values were calculated. Thus, this binding assay reflected the ability of the compound to bind with ergosterol.

### 2.9. Checkerboard Assay

A checkerboard microtiter test was performed to evaluate the interaction of coumarin derivative, 7-hydroxy-6-nitro-2H-1-benzopyran-2-one, with the antifungal drugs (azoles and Amphotericin B) against* A. fumigatus*, ATCC 46913, and* A. flavus*, ATCC 16013. A series of 2-fold dilutions, in eight for each coumarin derivative and antifungal drug, were made in RPMI-1640 to obtain four times the final concentration being achieved in the microtiter well. Furthermore, 50 *μ*L of each dilution of coumarin derivative was added to the 96-well microtiter plates in the vertical direction, while 50 *μ*L of each dilution of antifungal drugs was added in the horizontal direction, so that various combinations of coumarin derivative and antifungal drugs could be achieved. In addition, 10 *μ*L of inoculum from the spore suspension (1.5 × 10^5^ CFU mL^−1^) was added to each well, and the plates were incubated at 30°C for 3 days.

In order to evaluate the activity of the combinations of drugs, fractional inhibitory concentration (FIC) indices were calculated as FIC^A^ + FIC^B^, where FIC^A^ and FIC^B^ represent the minimum concentrations inhibiting the fungal growth for drugs A and B, respectively: FIC^A^ = MIC^A^ combination/MIC^A^ alone and FIC^B^ = MIC^B^ combination/MIC^B^ alone. A mean FIC index was calculated based on the following equation: FIC index = FIC^A^ + FIC^B^.

In addition, the interpretation was made as follows: synergistic (<0.5), additivity (0.5–1.0), indifferent (>1), or antagonistic (>4) [23, 24].

### 2.10. Drug Susceptibility Test

The minimum inhibitory concentrations (MIC) of coumarin derivative, 7-hydroxy-6-nitro-2H-1-benzopyran-2-one (Cou-NO_2_), and antifungal drug were determined in RPMI-1640 by microdilution assay using spore suspension (1.5 × 10^5^ CFU mL^−1^) and a drug concentration range of 1024 to 2,5 *μ*g/mL (twofold serial dilutions) [24]. MIC was defined as the lowest concentration at which no growth was observed. For the evaluation of the coumarin derivative as a modulator of antifungal properties, MICs of the antifungal drugs were determined in the presence of Cou-NO_2_ (2 *μ*g/mL) and at subinhibitory concentrations (MIC/8); the plates were incubated for 3 days at 30°C [25].

### 2.11. Data Analysis

The results were expressed in mean ± SE. Statistical analyses were performed with *t*-test. *P* < 0.05 was considered significant.

## 3. Results and Discussion

The difficulty in treating infections caused by* Aspergillus* spp. is in part related to the rather small arsenal of antifungal agents currently in use. The secondary metabolites derived from plants or biosynthesis serve as important fields of research for new antifungal agents. The coumarin derivative, 7-hydroxy-6-nitro-2H-1-benzopyran-2-one (Cou-NO_2_), a biosynthetic compound, has been reported for its antifungal activity [9].

The minimum inhibitory concentration of the coumarin derivative (Cou-NO_2_) on the clinical strains was similar to that observed previously by our [9] group, however now achieving an MIC of 32 mg/mL against the clinical strains* A. fumigatus* LM 743 and LM 135 (Table 1).

This study also verified Cou-NO_2_ action against* Aspergillus* mycelial growth and spore germination (*A. fumigatus* ATCC 46913 and* A. flavus* ATCC 16013).

**Figure 2:**
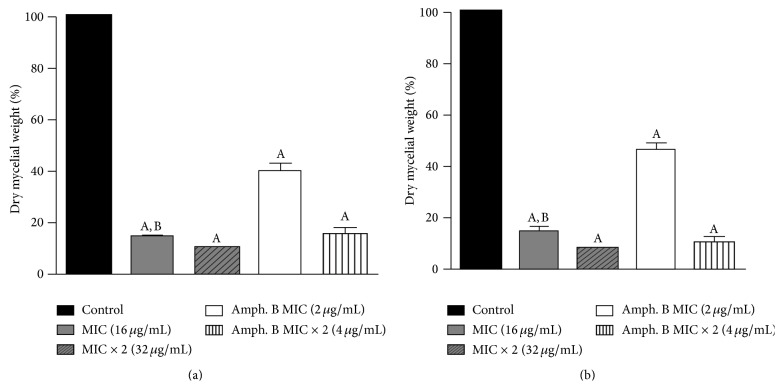
Percentage of dry mycelia weight produced by* A. fumigatus* (ATCC 46913) (a) and* A. flavus* (ATCC 16013) (b) in the absence (control) and presence of 7-hydroxy-6-nitro-2H-1-benzopyran-2-one (MIC: 16 *μ*g/mL; MIC × 2: 32 *μ*g/mL) and Amphotericin B (MIC: 2 *μ*g/mL; MIC × 2: 4 *μ*g/mL). Control produced 100% of dry mycelia weight. A: *P* < 0.05 compared to control. B: *P* < 0.05 compared to Amphotericin B with respective concentration.

The effect of differing concentrations of the test drug (MIC and MIC × 2) on mycelia growth was determined by measurement of mycelium dry weights, and the results are shown in Figure 2. With respect to effects on* A. fumigatus* ATCC 46913 and* A. flavus* ATCC 16013, it can be seen that Cou-NO_2_ in MIC concentrations of (16 *µ*g/mL) and MIC × 2 (32 *µ*g/mL) inhibited normal mycelia growth (*P* < 0.05) when compared to the control. The results show that Cou-NO_2_ at its MIC concentration was more potent than Amphotericin B at its respective MIC concentration (*P* < 0.05).

The production of hyphae and consequent mycelium formation are important virulence factors for* Aspergillus* spp.; Hyphae are more difficult to phagocytize and can induce apoptosis in macrophages, since they often form inside these cells after phagocytosis [26]. Thus, reductions in mycelia growth as an effect of the Cou-NO_2_ coumarin derivative interfering with the fungal virulence of* Aspergillus* spp. proved superior to Amphotericin B in its respective MIC concentrations.

*Aspergillus* spp. produce numerous asexual conidia which are spread throughout the environment and are also considered an important factor in triggering infections in the host. Thus, it is deemed important to quantitatively evaluate the power of a product to interfere with fungal spore germination [26].

**Figure 3:**
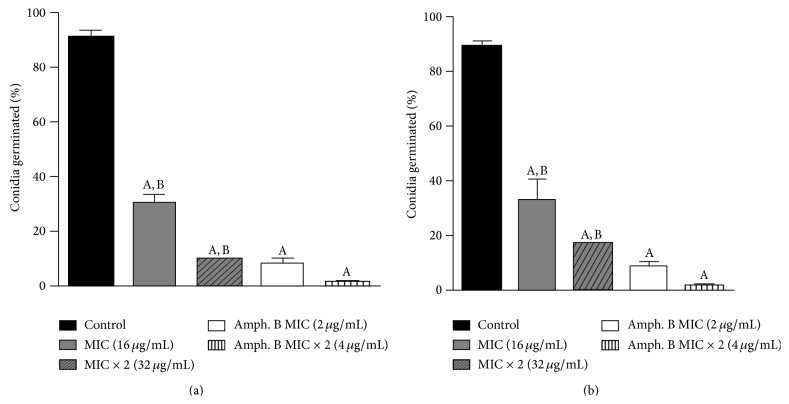
Percentage of conidial germination of* A. fumigatus* (ATCC 46913) (a) and* A. flavus* (ATCC 16013) (b) in the absence (control) and presence of 7-hydroxy-6-nitro-2H-1-benzopyran-2-one (MIC: 16 *μ*g/mL; MIC × 2: 32 *μ*g/mL) and Amphotericin B (MIC: 2 *μ*g/mL; MIC × 2: 4 *μ*g/mL). A: *P* < 0.05 compared to control. B: *P* < 0.05 compared to Amphotericin B with respective concentration.

The conidial percentage of* A. fumigatus* ATCC 46913 and* A. flavus* ATCC 16013 germinated in the presence and absence (control) of the test-drugs is shown in Figure 3. In the two test concentrations (MIC and MIC × 2), Cou-NO_2_ displayed significant inhibitory action for* Aspergillus* spp. (*P* < 0.05), as compared to the control. However, this action was shown to be less potent (*P* < 0.05) when compared with respective concentrations of Amphotericin B.

The great challenge when developing new antifungal drugs is in the similarity between fungal microorganisms cells and human cells. Thus, the desired targets for a new antifungal's action must be unique or at least sufficiently different from the host [27, 28]. Based on this, two important fungal structures become targets for detecting antifungal drugs: the fungal cell wall and ergosterol present in the plasma membrane.

Many drugs available for clinical use interact directly with ergosterol, causing damage to the fungal cell membrane [29]. If the effects of coumarin compounds on the fungal cell are due to ergosterol binding in the membrane, one can verify if they interact directly. In the presence of exogenous ergosterol in the culture medium, decreased binding of the product to the ergosterol of the membrane occurs. Thus, the product's MIC tends to increase in the presence of exogenous ergosterol, needing a much higher concentration to interact with the fungal membrane ergosterol [16, 21].

In this study, the MIC values for both Cou-NO_2_ experiments, with or without exogenous ergosterol in the culture media, were identical, suggesting that the coumarin derivative tested does not act via binding to ergosterol in the plasma membrane.

To investigate the action of the coumarin derivative (Cou-NO_2_) on the cell wall we carried osmotic shield testing with sorbitol; the test results are shown in Table 2. The MIC values of the Cou-NO_2_ increased 4-fold in the presence of sorbitol in the culture medium when compared to medium without sorbitol. This suggests that Cou-NO_2_ acts on the fungal cell wall structure. This is the first report of such activity on filamentous fungi.

According to Widodo et al. [30] coumarin acts by forming pores in the cell wall, with consequent release of cytoplasmic contents and cell death, confirming the results found in this study. However, other studies show that coumarins alter the morphology of the fungal mitochondrial cell and induce apoptosis [31, 32].

In addition to their inherent antimicrobial properties, natural products and their derivatives may alter the effects of standard antifungal agents (those used in clinical practice). The use of two or more antifungal combinations can lead to a reduction in the required drug dosages and decrease the normally produced adverse event profile [33, 34].

The addition of coumarin derivative, 7-hydroxy-6-nitro-2H-1-benzopyran-2-one, (Cou-NO_2_) to the growth medium at a subinhibitory concentration of 2 *μ*g/mL (MIC/8) resulted in a decreased MIC for Fluconazole 256 mg/mL to 128 mg/mL in both* A. flavus* and* A. fumigatus*. The MIC of Voriconazole decreased for* A. fumigatus *from4 *μ*g/mL to 0.5 *μ*g/mL and for* A. flavus* from 2 *μ*g/mL to 1 *μ*g/mL for the respective species. Also, the MIC of Itraconazole decreased from 128 *μ*g/mL to 32 *μ*g/mL, yet only for* A. fumigatus*, as demonstrated in Table 4. There was, however, no significant modulatory effect on the MIC of Amphotericin B (Table 3).

In Tables 4 and 5, the results are observed for combinations of the coumarin derivative 7-hydroxy-6-nitro-2H-1-benzopyran-2-one (Cou-NO_2_) with antifungal agents (Amphotericin B and azole derivatives) against* A. fumigatus* ATCC 46913 and* A. flavus* ATCC 16013. Additive effects were observed for the combinations of Cou-NO_2_ with Itraconazole and Voriconazole, resulting in a fractional inhibitory concentration (FIC) index of equal to 0.75 against both respective species tested. However, the combination of the Cou-NO_2_ and Amphotericin B and Fluconazole showed CIF index of 2 and 2, respectively, for each species tested.

According to these results, coumarin derivatives positively modulated the* in vitro* action of the azole derivatives, and the combinations with Voriconazole and Itraconazole obtained additive effects, suggesting future pharmacological use as an adjuvant for these drugs.

Several reports have been made concerning different antifungal combinations assayed* in vitro* and applied in the clinic [33, 35–37], and with other plant derivatives [10], but combinations of a coumarin derivative with synthetic drugs against* Aspergillus* spp. are reported here for the first time.

## 4. Conclusion

Based on these results, the present study demonstrates that 7-hydroxy-6-nitro-2 H-1-benzopyran-2-one (Cou-NO_2_) is capable of inhibiting both the mycelial growth and germination of conidia for the* Aspergillus* species tested, thus interfering in its virulence. The results also suggest that the action of coumarin derivatives affects the structure of the fungal cell wall. At subinhibitory concentration, Cou-NO_2_ enhanced the* in vitro* effects of azoles. In addition, in combination with the azoles (Voriconazole and Itraconazole) Cou-NO_2_ displays an additive effect. Thus, our studies support the potential use of 7-hydroxy-6-nitro-2H-1-benzopyran-2-one as an antifungal agent against* Aspergillus* species.

## Figures and Tables

**Table 1:** MIC values (*μ*g/mL) of 7-hydroxy-6-nitro-2H-1-benzopyran-2-one against clinical strains of *Aspergillus* spp.

Microorganisms	MIC values (*μ*g/mL)					
	*A*. *fumigatus *	*A*. *fumigatus *	*A*. *fumigatus *	*A*. *flavus *	*A*. *flavus *	*A*. *flavus *
	(LM 121)	(LM 135)	(LM 743)	(LM 35)	(LM 36)	(LM 23)
Cou-UNO_2_	16	32	32	16	16	16
Amphotericin B	2	2	2	2	2	2
Viability control	+	+	+	+	+	+
Negative control	−	−	−	−	−	−
Sensitivity control	+	+	+	+	+	+

*Note*. Cou-UNO_2_: 7-hydroxy-6-nitro-2H-1-benzopyran-2-one.

**Table 2:** MIC values (*μ*g/mL) of drugs in the absence and presence of sorbitol (0.8 M) and ergosterol (400 *μ*g/mL) against *Aspergillus* spp.

Microorganisms	MIC values (*μ*g/mL)					
	*A*. *fumigatus* ATCC 46913	*A*. *flavus* ATCC 16013	*A*. *fumigatus* ATCC 46913	*A*. *flavus* ATCC 16013	*A*. *fumigatus* ATCC 46913	*A*. *flavus* ATCC 16013
Drugs	−Sterols		+Sorbitol		+Ergosterol	
Cou-UNO_2_	16	16	256	256	16	16
Amphotericin B	2	2	—	—	16	16
Fluconazole	256	512	512	512	256	512

*Note*. Cou-UNO_2_: 7-hydroxy-6-nitro-2H-1-benzopyran-2-one.

**Table 3:** MIC values of antifungals in the absence and presence of coumarin derivative, 7-hydroxy-6-nitro-2H-1-benzopyran-2-one, MIC/8, against *Aspergillus* spp.

Drugs	MIC alone		Combined MIC (Cou-NO_2_)	
	*A*. *fumigatus* ATCC 46913	*A*. *flavus* ATCC 16013	*A*. *fumigatus* ATCC 46913	*A*. *flavus* ATCC 16013
Amphotericin B	2	2	2	2
Fluconazole	256	256	128	128
Itraconazole	128	128	32	128
Voriconazole	4	2	0,5	1

**Table 4:** MIC of Antifungal drugs and effect of combination with coumarin derivative, 7-hydroxy-6-nitro-2H-1-benzopyran-2-one (Cou-UNO_2_), against *A*. *flavus*, ATCC 16013.

Antifungal + Cou-UNO_2_	MIC	FIC index
	(*μ*g/mL)	(Type of interaction)
Cou-UNO_2_	16	
Amphotericin B	2	
Fluconazole	256	
Itraconazole	128	
Voriconazole	4	
Cou-UNO_2_/Amphotericin B	16/2	2 (Indifferent)
Cou-UNO_2_/Fluconazole	16/256	2 (Indifferent)
Cou-UNO_2_/Itraconazole	8/32	0,75 (Additivity)
Cou-UNO_2_/Voriconazole	8/1	0,75 (Additivity)

*Note*. Cou-UNO_2_: 7-hydroxy-6-nitro-2H-1-benzopyran-2-one. FIC: fractional inhibitory concentration. MIC: minimal inhibitory concentration.

**Table 5:** MIC of antifungal drugs and effect of combination with coumarin derivative, 7-hydroxy-6-nitro-2H-1-benzopyran-2-one (Cou-1), against *A*. *fumigatus*, ATCC 46913.

Antifungal + Cou-UNO_2_	MIC	FIC index
	(*μ*g/mL)	(Type of interaction)
Cou-UNO_2_	16	
Amphotericin B	2	
Flucanozole	256	
Itraconazole	128	
Voriconazole	2	
Cou-UNO_2_/Amphotericin B	16/2	2 (indifferent)
Cou-UNO_2_/Flucanozole	16/256	2 (indifferent)
Cou-UNO_2_/Itraconazole	4/64	0,75 (additivity)
Cou-UNO_2_/Voriconazole	8/0,5	0,5 (additivity)

*Note*. Cou-UNO_2_: 7-hydroxy-6-nitro-2H-1-benzopyran-2-one. FIC: fractional inhibitory concentration. MIC: minimal inhibitory concentration.
